# Role of EZH2-mediated H3K27me3 in placental ADAM12-S expression: implications for fetoplacental growth

**DOI:** 10.1186/s12916-022-02391-4

**Published:** 2022-05-25

**Authors:** Ya-nan Zhu, Xiao-wen Gan, Fan Pan, Xiao-tian Ni, Leslie Myatt, Wang-sheng Wang, Kang Sun

**Affiliations:** 1 Center for Reproductive Medicine, Ren Ji Hospital, Shanghai Jiao Tong University School of Medicine, Shanghai, People’s Republic of China; 2 Shanghai Key Laboratory for Assisted Reproduction and Reproductive Genetics, Shanghai, People’s Republic of China; 3 Department of Obstetrics and Gynecology, East Hospital, Tongji University School of Medicine, Shanghai, People’s Republic of China; 4 Department of Obstetrics and Gynecology, Oregon Health & Science University, Portland, USA

**Keywords:** ADAM12, EZH2, IGFBP, IGF, Placenta, Fetus

## Abstract

**Background:**

Enhancer of zeste homolog 2 (EZH2)-mediated histone 3 lysine 27 trimethylation (H3K27me3) is a transcription silencing mark, which is indispensable for cell lineage specification at the early blastocyst stage. This epigenetic repression is maintained in placental cytotrophoblasts but is lifted when cytotrophoblasts differentiate into syncytiotrophoblasts. However, the physiological impact of this lift remains elusive. Here, we investigated whether lifting EZH2-mediated H3K27me3 during syncytialization upregulates the expression of a short secretory isoform of a disintegrin and metalloprotease 12 (ADAM12-S), a well-recognized placenta-derived protease that cleaves insulin-like growth factor binding protein 3 to increase insulin-like growth factor (IGF) bioavailability for the stimulation of fetoplacental growth. The transcription factor and the upstream signal involved were also explored.

**Methods:**

Human placenta tissue and cultured primary human placental cytotrophoblasts were utilized to investigate the role of EZH2-mediated H3K27me3 in ADAM12-S expression and the associated transcription factor and upstream signal during syncytialization. A mouse model was used to examine whether inhibition of EZH2-mediated H3K27me3 regulates placental ADAM12-S expression and fetoplacental growth.

**Results:**

EZH2 and ADAM12 are distributed primarily in villous cytotrophoblasts and syncytiotrophoblasts, respectively. Increased ADAM12-S expression, decreased EZH2 expression, and decreased EZH2/H3K27me3 enrichment at the *ADAM12* promoter were observed during syncytialization. Knock-down of EZH2 further increased ADAM12-S expression in trophoblasts. Syncytialization was also accompanied by increased STAT5B expression and phosphorylation as well as its enrichment at the *ADAM12* promoter. Knock-down of STAT5B attenuated ADAM12-S expression during syncytialization. Epidermal growth factor (EGF) was capable of inducing ADAM12-S expression via stimulation of STAT5B expression and phosphorylation during syncytialization. Mouse studies revealed that administration of an EZH2 inhibitor significantly increased ADAM12-S levels in maternal blood and fetoplacental weights along with decreased H3K27me3 abundance and increased ADAM12-S expression in the placenta.

**Conclusions:**

Lifting EZH2-mediated H3K27me3 increases ADAM12-S expression during syncytialization with the participation of EGF-activated STAT5B, which may lead to elevation of ADAM12-S level in maternal blood resulting in increased IGF bioavailability for the stimulation of fetoplacental growth in pregnancy. Our studies suggest that the role of EZH2-mediated H3K27me3 may switch from cell lineage specification at the early blastocyst stage to regulation of fetoplacental growth in later gestation.

## Background

Epigenetic modification of histones plays an important role in gene expression. Among histone epigenetic marks, enhancer of zeste homolog 2 (EZH2)-mediated trimethylation of histone 3 at lysine27 (H3K27me3) is a well-recognized transcription silencing mark [1]. Lifting this repressive mark will loosen chromatin compaction so that the associated gene promoter can be accessed by transcription factors to drive its expression [1]. Previous studies have demonstrated that EZH2-mediated H3K27me3 is indispensable for cell lineage specification at the early blastocyst stage [2, 3]. However, its role in later gestation remains elusive. Our previous study has shown that EZH2-mediated H3K27me3 is lifted during syncytialization of placental trophoblasts, which increases the expression of 11β-hydroxysteroid dehydrogenase 2 (11β-HSD2) [4], a glucocorticoid inactivating enzyme known as a placental barrier offering protection for fetal growth from maternal glucocorticoids [5]. This finding suggests that the role of EZH2-mediated H3K27me3 in the placenta may have changed in later gestation to regulate gene expression pertinent to fetal growth. Given the relatively broad range of EZH2-regulated gene expression, we proposed that there might be additional fetoplacental growth-pertinent genes which are subject to the regulation by EZH2-mediated H3K27me3 in the placenta.

It is known that insulin-like growth factors (IGFs), IGF-1, and IGF2, are crucial stimulators of fetoplacental growth [6–9]. However, IGFs are normally sequestered from IGF receptor type 1 (IGF-1R) by insulin-like growth factor binding proteins (IGFBPs) [10, 11]. There are six distinct IGFBPs which differ in molecular mass, post-translational modification, and binding affinity [12]. Among them, IGFBP3 is the most abundant isoform in the circulation and binds over 95% of circulating IGFs [10, 13, 14]. Therefore, proteolytic cleavage of IGFBP3 is essential for IGF bioavailability to maintain fetoplacental growth in pregnancy. It is now recognized that the short secretory isoform of a disintegrin and metalloprotease 12 (ADAM12-S) is a very important protease for IGFBP3 cleavage [15–17]. In pregnancy, there is a marked increase in ADAM12-S level in maternal blood [17–21], which reconciles with the findings that the IGFBP-3 protease in the serum of pregnant women is sensitive to ADAM12-S-specific inhibitors, and the degraded fragments of IGFBP-3 found in pregnancy serum are similar in molecular weight to those cleaved by ADAM12-S [16–18, 22, 23]. These lines of evidence indicate that IGFBP3 cleavage in maternal blood is attributed largely to ADAM12-S in pregnancy. It is believed that ADAM12-S in maternal blood derives primarily from the placenta [17]. In the placental villi, ADAM12-S was found to be localized mainly in the syncytial layer but to a lesser extent in its progenitor cytotrophoblasts [24], suggesting that there is up-regulation of ADAM12-S expression during syncytialization of cytotrophoblasts resulting in increased ADAM12-S production from the placenta in pregnancy. Our preliminary study demonstrated that syncytialization of cytotrophoblasts was indeed accompanied by increased ADAM12-S expression, which was further increased by an EZH2 inhibitor, suggesting that EZH2-mediated H3K27me3 may also play a role in the regulation of ADAM12-S expression during syncytialization.

STAT5B is a member of the signal transducer and activator of transcription (STAT) family, which mediates the transcription of numerous genes involved in cell proliferation, differentiation and survival upon phosphorylation. A variety of growth stimuli, including epidermal growth factor (EGF), growth hormone and prolactin, utilize STAT5B as a transcription factor [25–27]. In silico analysis revealed multiple putative STAT5B binding sites in the promoter region of the *ADAM12* gene, suggesting that STAT5B may be a transcription factor driving ADAM12-S expression. In this context, we hypothesized that EZH2-mediated H3K27me3 repressed ADAM12-S expression in cytotrophoblasts, and this repression was lifted during syncytialization resulting in increased ADAM12-S expression with the participation of STAT5B as a transcription factor upon activation by a growth factor. Consequently, increased ADAM12-S output from the placenta is achieved in pregnancy so that IGFBP3 cleavage and IGF bioavailability are enhanced to stimulate fetoplacental growth. Herein, we examined the hypothesis by using human placental villous tissue and an in vitro syncytialization model of primary human placental trophoblasts as well as an in vivo mouse model.

## Methods

### Collection of human placental tissue

Human placental villous tissue was collected from uncomplicated pregnancies either at first trimester termination of pregnancy (6 to 8 weeks) or at term (38 to 40 weeks) elective cesarean section with written informed consent under a protocol approved by the Ethics Committee of Ren Ji Hospital, School of Medicine, Shanghai Jiao Tong University.

### Immunofluorescent and immunohistochemical staining of human placental villous tissue

To examine the distribution of EZH2, ADAM12, STAT5B, and phosphorylated STAT5B in the villi, villous tissues from both first trimester and term pregnancies were fixed with formalin and embedded in paraffin. After deparaffination, rehydration, and inactivation of endogenous peroxidase, antigen retrieval was performed by boiling the tissue section in sodium citrate solution. For immunofluorescent staining, the section was permeabilized with 0.4% Triton X-100 in phosphate buffer solution (PBS). After blocking with normal goat serum (Proteintech, Wuhan, China, #B900780), the primary antibody against EZH2 (Cell Signaling, Danvers, MA, #5246) or ADAM12 (Abcam, Cambridge, MA, #223476) or 11β-HSD2 (Santa Cruz Biotechnology, Santa Cruz, CA, sc- 365529) was applied to the section at 1:100 dilution for incubation overnight at 4 °C, followed by incubation with fluorescein isothiocyanate- or Alexa Fluor 594-labeled or Alexa Fluor 488-labeled secondary antibody (Proteintech) for 1.5 hrs at room temperature. Non-immune serum instead of primary antibodies was applied as negative control. Nuclei were stained with DAPI (1 μg/mL). The fluorescence signals were examined under a fluorescence microscope (Zeiss, Oberkochen, Germany).

For immunohistochemical staining, endogenous peroxidase activity was quenched with 0.3% H_2_O_2_. After blocking with normal horse serum (Vector Laboratories, Burlingame, CA), primary antibodies against EZH2 (Cell Signaling, #5246), ADAM12 (Abcam, #ab223476), STAT5B (Abcam, #17891), and phosphorylated STAT5B at Tyr694 (Cell Signaling, #9351) were applied at 1:200 dilution for incubation overnight at 4 °C. Pre-immune serum instead of the primary antibody was applied for negative control. The antibody against ADAM12 recognizes both ADAM12-S and a long transmembrane isoform of ADAM12. Following washing with PBS (phosphate buffer solution), corresponding secondary antibodies conjugated with biotinylated horseradish peroxidase H (Vector Laboratories) were applied to the section to incubate for 1 hr, and red color was developed by adding 3-amino-9-ethyl carbazole (Vector Laboratories) as substrate. The section was counterstained with hematoxylin and examined under a regular microscope (Zeiss).

### Isolation and culture of primary human placental trophoblasts

Villous cytotrophoblasts were isolated from term placenta using a modification of Kliman’s method as described previously [28]. Briefly, pieces of villous tissue were cut from the maternal side of the placenta and washed with PBS to remove residual blood. The tissue was minced prior to digestion with 0.125% trypsin (Sigma Chemical Co, St Louis, MO) and 0.03% deoxyribonuclease I (Sigma) in Dulbecco’s modified Eagle’s medium (DMEM) (Gibco, Grand Island, NY) containing 1% antibiotics (Gibco) at 37 °C. Digested cells were purified by centrifugation on discontinuous Percoll® density gradients (5–65%) (GE Healthcare Bio-Sciences, Uppsala, Sweden). Cytotrophoblasts between density markers of 1.049 and 1.062 g/ml were collected and cultured at 37 °C in 5% CO_2_/95% air in DMEM containing 10% fetal bovine serum (FBS) (Gibco) and 1% antibiotics (Gibco) to allow spontaneous syncytialization in vitro. The process of syncytialization (3, 24, and 48 hrs after plating) was visualized under a microscope after staining with hematoxylin–eosin (HE). For examination of the expression of syncytialization markers, cultured trophoblasts were harvested for total RNA extraction at 3 and 48 hrs after plating. After reverse transcription, quantitative real-time polymerase chain reaction (qRT-PCR) was applied to measure the changes of syncytialization markers including *CGB3*, *GCM1*, *ERVW-1*, and *CDH1,* the genes encoding β subunit of human chorionic gonadotropin (β-hCG), glial cells missing transcription factor 1, syncytin-1 and E-cadherin respectively with the following primers: *CGB3*, 5′-GCTCACCCCAGCATCCTATC (forward) and 5′-CCTGGAACATCTCCATCCTTG (reverse); *GCM1*, 5′-GACCAGGTCTCTTCCAGGTG (forward) and 5′-ATTTGCTGCTCTTGCTTGGC (reverse); *ERVW-1*, 5′-CTACCCCAACTGCGGTTAAA (forward) and 5′-GGTTCCTTTGGCAGTATCCA (reverse); *CDH1*, 5′-CTGCCAATCCCGATGAAATTG (forward) and 5′-TCCTTCATAGTCAAACACGAGC (reverse). The methods of RNA extraction, reverse transcription, and qRT-PCR were detailed below in the corresponding section.

### Examination of the changes of EZH2, ADAM12-S, STAT5B, and EGF receptor during syncytialization

To observe changes of EZH2, ADAM12-S, STAT5B, phosphorylated STAT5B, and EGF receptor (EGFR) abundance during syncytialization, isolated cytotrophoblasts were cultured for 3, 24, or 48 hrs to allow spontaneous syncytialization. RNA and protein were extracted from the cells at the above time points for determination of the abundance of EZH2, ADAM12-S, STAT5B, phosphorylated STAT5B, and EGFR with qRT-PCR or Western blotting. In another set of experiments, culture medium of the trophoblasts was replaced with serum-free medium at 3, 24, or 48 hrs for further incubation for 12 h. The conditioned medium was then collected for measurement of secreted ADAM12-S during syncytialization with Western blotting.

The role of EZH2 and STAT5B in the regulation of ADAM12-S expression during syncytialization was studied by transfecting isolated cytotrophoblasts with small interfering RNA (siRNA) against EZH2 or STAT5B immediately after isolation using lipofectamine RNAiMAX reagent (Invitrogen, San Diego, CA). Sequences of siRNA are as follows: EZH2, GAGGGAAAGUGUAUGAUAATT (sense) and UUAUCAUACACUUUCCCUCTT (antisense) (Gene Pharma Co., Ltd., Shanghai, China); STAT5B, CUCAGUAGAUCUUGAUAAUTT (sense) and AUUAUCAAGAUCUACUGAGTT (antisense) (Gene Pharma Co., Ltd.) Randomly scrambled siRNA served as a negative control. After transfection for 48 hrs, the cells were collected for RNA and protein extraction for determination of ADAM12-S, EZH2, and STAT5B abundance with qRT-PCR and Western blotting. The efficiencies of EZH2 knock-down were 65% and 60% at mRNA and protein levels respectively, and the efficiencies of STAT5B knock-down were 85% and 90% at mRNA and protein levels, respectively.

To observe the time course of EGF on STAT5B phosphorylation, syncytiotrophoblasts were treated with EGF (10 ng/mL, Peprotech, East Windsor, NJ) for 5, 10, 20, and 40 mins, and cellular protein was then extracted for measurement of STAT5B phosphorylation with Western blotting. The role of STAT5B in the induction ADAM12-S by EGF was studied by treating syncytiotrophoblasts with EGF (10 ng/mL) for 24 hrs in the presence or absence of siRNA-mediated knock-down of STAT5B expression.

### Extraction of RNA and analysis with qRT-PCR

Total RNA was extracted from cultured trophoblasts using an RNA isolation kit (Foregene, Chengdu, China). After determination of RNA purity and concentration with NanoDrop ND-2000 (Thermo Fisher Scientific, Carlsbad, CA), mRNA was reversely transcribed to complementary DNA (cDNA) using a PrimeScript RT Master Mix Perfect Real-Time Kit (Takara, Kyoto, Japan). The abundance of *ADAM12S*, *EZH2*, and *STAT5B* mRNA was determined with qRT-PCR using the above-transcribed cDNA and power Sybr Premix Ex TaqTM (Takara) following a previously described protocol [29]. Relative mRNA abundance was quantified using the 2^−△△Ct^ method and normalized to glyceraldehyde 3-phosphate dehydrogenase (*GAPDH*). Primers used for qRT-PCR were as follows: *ADAM12-S*, 5′-GTGACAAGTTTGGCTTTGGAG (forward) and 5′-GTGAGGCAGTAGACGCATG (reverse); *EZH2*, 5′-AAGCAGGGACTGAAACGG (forward) and 5′-TGAGGCTTCAGCACCACT (reverse); *STAT5B*, 5′-GTCCCTGAGTTTGTGAACGC (forward) and 5′-CCAGATCGAAGTCCCCATCG (reverse); *GAPDH*, 5′-CCCCTCTGCTGATGCCCCCA (forward) and 5′-TGACCTTGGCCAGGGGTGCT (reverse).

### Extraction of protein and analysis with Western blotting

Cellular protein was extracted with radioimmunoprecipitation assay (RIPA) lysis buffer (Active Motif, Carlsbad, CA) containing a protease inhibitor cocktail (Roche, Basel, Switzerland) and a phosphatase inhibitor (Roche). After the determination of protein concentration with the Bradford assay, the standard protocol of Western blotting was followed to determine the protein abundance of EZH2, STAT5B, phosphorylated STAT5B, and ADAM12-S. Briefly, 20–30 μg protein was electrophoresed in 9% sodium dodecyl-sulfate (SDS)-polyacrylamide gel and transferred to the nitrocellulose membrane (Merck Millipore, Darmstadt, Germany). After blocking with 5% nonfat milk, the membrane was probed with primary antibodies against EZH2 (1:2000, Cell Signaling, #5246), STAT5B (1:250, Invitrogen, #712500), phosphorylated STAT5B at Tyr694 (1:1000, Cell Signaling, #9351), ADAM12-S (1:1000, Abcam, #223476) and GAPDH (1:10,000, Proteintech, #60004–1) respectively overnight at 4 °C. After washing with 1 × TBST (Tween 20/tris-buffered salt solution), the membrane was incubated with horseradish peroxidase-﻿conjugated secondary antibody (Proteintech) for 1 hr. The bands with peroxidase activity were detected with a chemiluminescence detection system (Merck Millipore) and visualized using a G-Box chemiluminescence image capture system (Syngene, Cambridge, UK). The ratio of band densities of target protein over GAPDH was used to indicate the relative abundance of target protein. ﻿

The abundance of ADAM12-S in the conditioned culture medium was determined using a concentrated culture medium prepared with a centrifugal filter device following instructions provided by the manufacturer (Merck Millipore). Briefly, after rinsing the filter device with 0.5 ml ultrapure water, 0.5 ml conditioned medium was placed in the filter and centrifuged at 4000* g* at 4 °C for 18 mins. After centrifugation, the residual culture medium was desalted with ultrapure water by centrifugation, and then the concentrated culture medium on top of the filter was collected for analysis with Western blotting. The abundance of ADAM12-S in the conditioned medium was expressed as the ratio of band density of ADAM12-S over that of the most prominent protein band stained by Ponceau S.

### Chromatin immunoprecipitation (ChIP) assay

ChIP assay was carried out to examine the enrichment of EZH2, H3K27me3, and STAT5B at *ADAM12* promoter. Details of ChIP assay procedures have been described previously [30]. In brief, trophoblasts before and after syncytialization (3 and 48 hrs after plating, respectively) were fixed with 1% formaldehyde for 10 mins. Fixation was terminated by incubating the cells with 125 mM glycine for 5 mins. The cells were then lysed with 1% SDS lysis buffer and sonicated to shear chromatin DNA to a size around 500 bp. Sheared DNA was immunoprecipitated with the primary antibody against EZH2 (Active Motif, #39901) or H3K27me3 (Active Motif, #39155) or STAT5B (Invitrogen, #135300). The precipitated immune complex was pulled down with protein A + G agarose magnetic beads (Merck Millipore). An equal amount of pre-immune IgG instead of the specific antibody served as a negative control. Sheared DNA without immunoprecipitation served as input control. After washing, reverse cross-linking of the immune complex was performed in 5 M NaCl at 65 °C overnight. RNA contamination was removed by incubation with ribonuclease A for 30 mins and the protein in the complex was digested by incubation with proteinase K at 45 °C for 1 hr. Both input and immunoprecipitated DNA were extracted using a DNA purification kit (Cwbiotech, Beijing, China) for subsequent qRT-PCR with primers aligning the putative binding site of EZH2/H3K27me3 or STAT5B at *ADAM12* promoter. The primer sequences for qRT-PCR were as follows: EZH2/H3K27me3 binding site in *ADAM12* promoter: 5′-GCCCGGAACAAGGATGAGAA (forward) and 5′-TAAGAAGCACCTGGGTTGGC (reverse); STAT5B binding site in *ADAM12* promoter: 5′-AGCCATCCTCCATAGCTCCA (forward) and 5′-TTCTCATCCTTGTTCCGGGC (reverse). The data were analyzed using the following equation to indicate the enrichments of EZH2, H3K27me3 or STAT5B at the promoter of *ADAM12* gene: percent input = 100% × 2^[(Ct (input sample)−Ct (IP sample)^.

### Animal study

The mouse study was approved by the Institutional Review Board of Ren Ji Hospital, School of Medicine, Shanghai Jiao Tong University, and followed ARRIVE guidelines. C57BL/6 mice were purchased from the Charles River Company (Beijing, China) with accreditation from AAALAC (Association for Assessment and Accreditation of Laboratory Animal Care) International. Mice aged 10 to 13 weeks were mated and gestational day (GD) was counted as 0.5 when a vaginal plug was present. Pregnant mice were transferred to individual cages and divided randomly into control (*n* = 9) and EPZ (*n* = 8) groups. The information of each dam was given in Table 1. The EPZ group was injected with an EZH2 inhibitor EPZ005687 (Selleckchem, Houston, TX) (8 mg/kg body weight) intraperitoneally once a day from GD12.5 to 17.5, while the control group received an equal volume of solvent (2% dimethyl sulphoxide in normal saline). The dam was sacrificed by cervical dislocation on GD 18.5 and maternal blood was collected from the orbital sinus. The fetuses and placentas were quickly removed and weighed. After weighing, the placenta was frozen in liquid nitrogen and stored at − 80 °C for later RNA and protein extraction after homogenization with an electric homogenizer (Biheng Bio., Shanghai, China). The fetus sex was determined by the distance between the anus and genitalia as well as by measuring the unique genes (*Sry* and *Ssty1*) encoded in the Y chromosome with qRT-PCR with primers as follows: *Sry*, 5′- ATGTCAAGCGCCCCATGAAT (forward) and 5′- CCCTCCGATGAGGCTGATATTTA (reverse); *Ssty1*, 5′- AGGTTTTGCCTCCCATAGTAGT (forward) and 5′- CCCCTCCAGTTGACCTCAG (reverse). Placental *Adam12* and *Hsd11b2* mRNA abundance was measured with qRT-PCR. The relative mRNA abundance of *Adam12* and *Hsd11b2* was quantified using the 2^−△△Ct^ method and normalized to *Gapdh*. Primers used for qRT-PCR were as follows: *Adam12s*, 5′-TGTGGAAATGGCTATGTGGA (forward) and 5′-CAGGTGGTAGCGTTACAGCA (reverse); *Hsd11b2*, 5′-CAGAGGACATCAGCCGTGTTCT (forward) and 5′-GAAAGTCGCCACTGGAGACAGT (reverse); *Gapdh*, 5′-AGGTCGGTGTGAACGGATTTG (forward) and 5′-TGTAGACCATGTAGTTGAGGTCA (reverse). Placental H3K27me3 abundance was measured with Western blotting using an antibody against H3K27me3 (1:1000, Active motif, #39155). Internal loading control was examined by probing the blot with a H3 antibody (1:5000, Abcam, #1791). ADAM12-S in the maternal blood was measured with an ELISA kit (MyBioSourse, San Diego, CA, #MBS177214).

**Table 1:** Maternal weight, litter size, and offspring sex in mice

	No	Weight (g)	Litter size	Offspring sex (Female/male)
Control	1	30.0	6	5/1
	2	33.6	9	4/5
	3	30.0	7	2/5
	4	32.9	7	1/6
	5	30.4	6	3/3
	6	32.6	7	3/4
	7	35.0	7	4/3
	8	33.8	7	5/2
	9	32.4	7	5/2
EPZ	1	29.9	5	1/4
	2	30.7	7	4/3
	3	36.0	7	3/4
	4	32.5	7	5/2
	5	33.5	8	5/3
	6	34.0	7	4/3
	7	33.7	6	4/2
	8	33.0	6	3/3
*P* value		0.508	0.399	0.839

### Statistical analysis

All data are expressed as mean ± standard error of mean (SEM). Shapiro–Wilk normality test was used to examine the normal distribution of the data. Unpaired or paired Student’s *t*-test was employed for comparison between two groups with normal distribution. Mann–Whitney *U* test was used for unpaired data of two groups when the data were not normally distributed. One-way ANOVA test followed by Newman-Keuls multiple comparisons test was performed to analyze paired data of more than two groups with normal distribution. Significance was set at *P* < 0.05.

## Results

### Distinct distribution of EZH2 and ADAM12 in cytotrophoblasts and syncytiotrophoblasts of placental villi

**Fig. 1:**
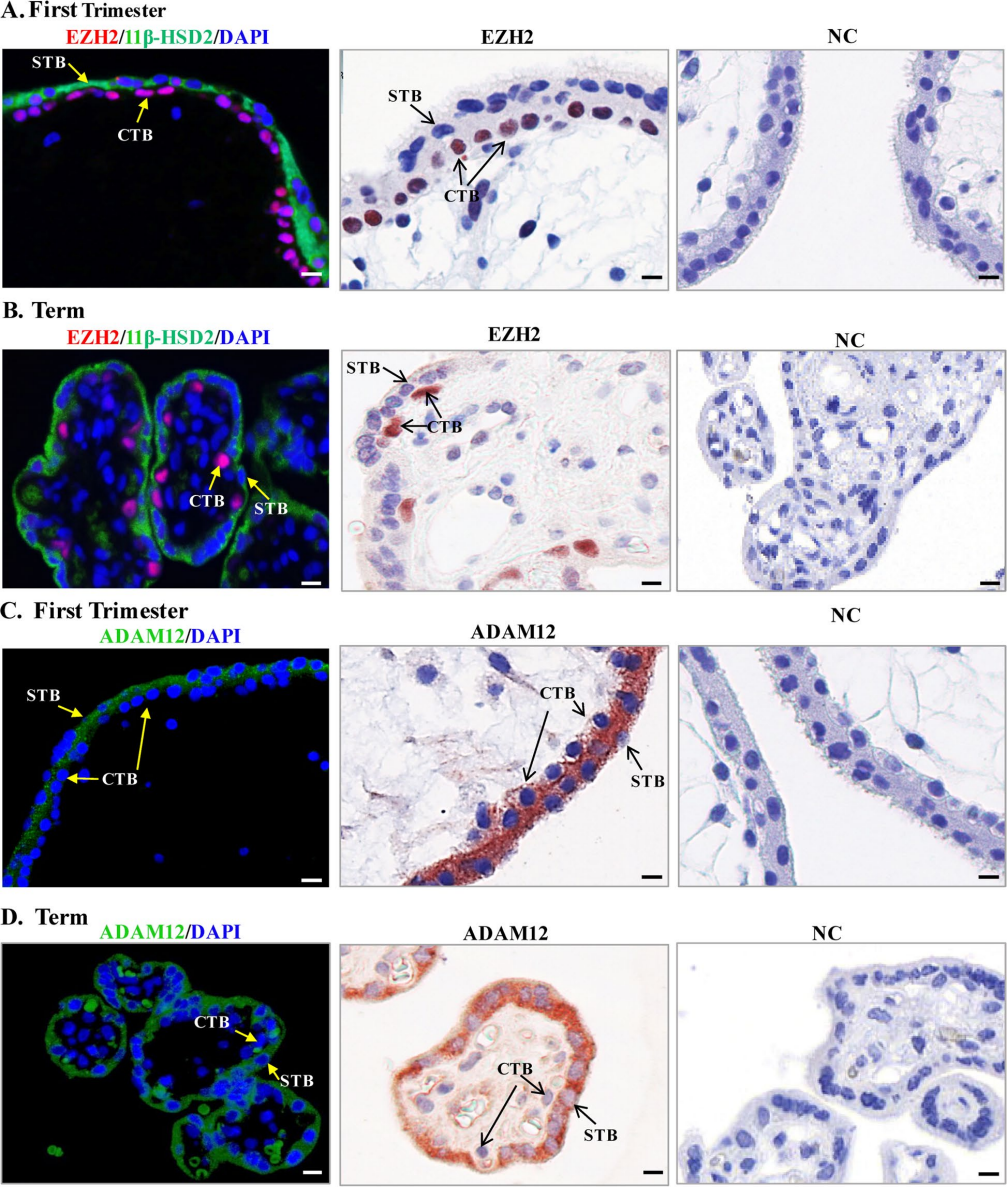
Distinct distribution of EZH2 and ADAM12 in human placental villi. **A**, **B** Immunofluorescent staining showed that EZH2 (red) was localized mainly in the cytotrophoblast (CTB) but not the syncytiotrophoblast (STB) which was stained for 11β-HSD2 (green) at both early and term pregnancies. Nuclei were counterstained blue with DAPI. Immunohistochemical staining revealed a similar distribution pattern of EZH2. **C**, **D** Immunofluorescent staining showed that ADAM12 (green) was mainly localized in the syncytiotrophoblast (STB) and to a much lesser extent in the cytotrophoblast (CTB) at both early and term pregnancies. Nuclei were counterstained blue with DAPI. Immunohistochemical staining revealed a similar distribution pattern ADAM12. Non-immune serum served as negative control (NC). Scale bars, 20 μm

Immunofluorescence and immunohistochemical staining revealed a distinct pattern of EZH2 and ADAM12 distribution in the chorionic villi of the human placenta (Fig. 1). EZH2 was localized primarily in the cytotrophoblasts at both early and term gestation, which was in marked contrast to the localization of 11β-HSD2, a well-described enzyme in the syncytial layer (Fig. 1A, B). In contrast to EZH2, ADAM12 staining was found mainly in the syncytial layer and a weak ADAM12 signal was detected in the cytotrophoblasts at both early and term gestation (Fig. 1C, D).

### Role of EZH2-mediated H3K27me3 in the regulation of ADAM12-S expression during syncytialization

**Fig. 2:**
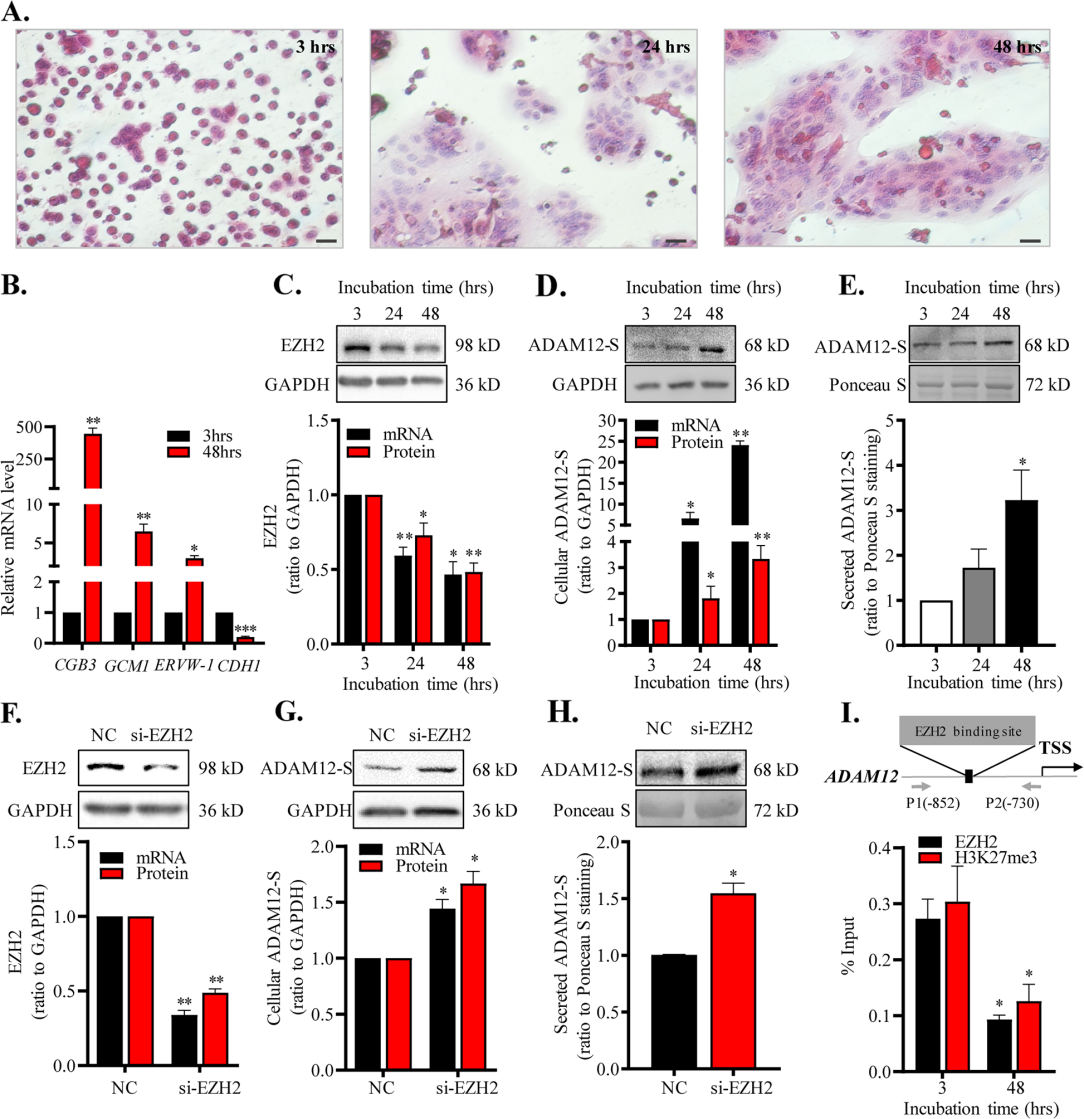
Role of EZH2-mediated H3K27me3 in the regulation of ADAM12-S expression during syncytialization of human trophoblasts. **A** HE staining showed the fusion of mononuclear cytotrophoblasts into multinuclear syncytiotrophoblasts with incubation time. Scale bars, 20 μm. **B** Increased *CGB3*, *GCM1*, *ERVW-1* and decreased *CDH1* mRNA abundance, the syncytialization markers, in cultured trophoblasts upon syncytialization (*n* = 4). **C** Decreased *EZH2* mRNA (*n* = 4) and protein (*n* = 6) abundance during syncytialization. **D**, **E** Increased *ADAM12S* mRNA (*n* = 4) and protein (*n* = 5) abundance in trophoblasts and secreted ADAM12-S abundance in the culture medium (*n* = 5) of trophoblast during syncytialization. **F**–**H** siRNA-mediated knockdown of EZH2 expression increased the abundance of *ADAM12S* mRNA (*n* = 3) and protein (*n* = 4) in trophoblasts as well as secreted ADAM12-S protein in the culture medium of trophoblasts (*n* = 3). Randomly scrambled siRNA served as negative control (NC). Top panels of **C**–**H** are representative immunoblots. **I** Decreased enrichment of EZH2 and H3K27me3 at *ADAM12* promoter upon syncytialization of cultured trophoblasts (*n* = 3). Top panel of I illustrates aligning positions of primers used in the ChIP assay. TSS, transcription start site. Data are means ± SEM. *P < 0.05, **P < 0.01 vs 3 hrs or NC

HE staining of cultured primary human placental trophoblasts showed that mononuclear cytotrophoblasts fused progressively into multinuclear syncytiotrophoblasts with incubation time (Fig. 2A) along with dramatic increases in *CGB3*, *GCM1*, *ERVW-1* mRNA abundance and a decrease in *CDH1* mRNA abundance, the well-described syncytialization markers (Fig. 2B). Analyses with qRT-PCR and Western blotting showed that the abundance of *EZH2* mRNA and protein in trophoblasts was significantly decreased (Fig. 2C) while the abundance of *ADAM12S* mRNA and protein in trophoblasts and secreted ADAM12-S in the culture medium of trophoblast was significantly increased during syncytialization (Fig. 2D, E). Moreover, siRNA-mediated knock-down of EZH2 expression significantly increased *ADAM12S* mRNA and protein abundance in trophoblasts and secreted ADAM12-S in the culture medium of trophoblasts (Fig. 2F–H). ChIP assay revealed that the enrichment of EZH2 and H3K27me3 at *ADAM12* promoter was significantly decreased upon syncytialization (Fig. 2I).

### Role of EGF/STAT5B pathway in the induction of ADAM12-S expression during syncytialization

**Fig. 3:**
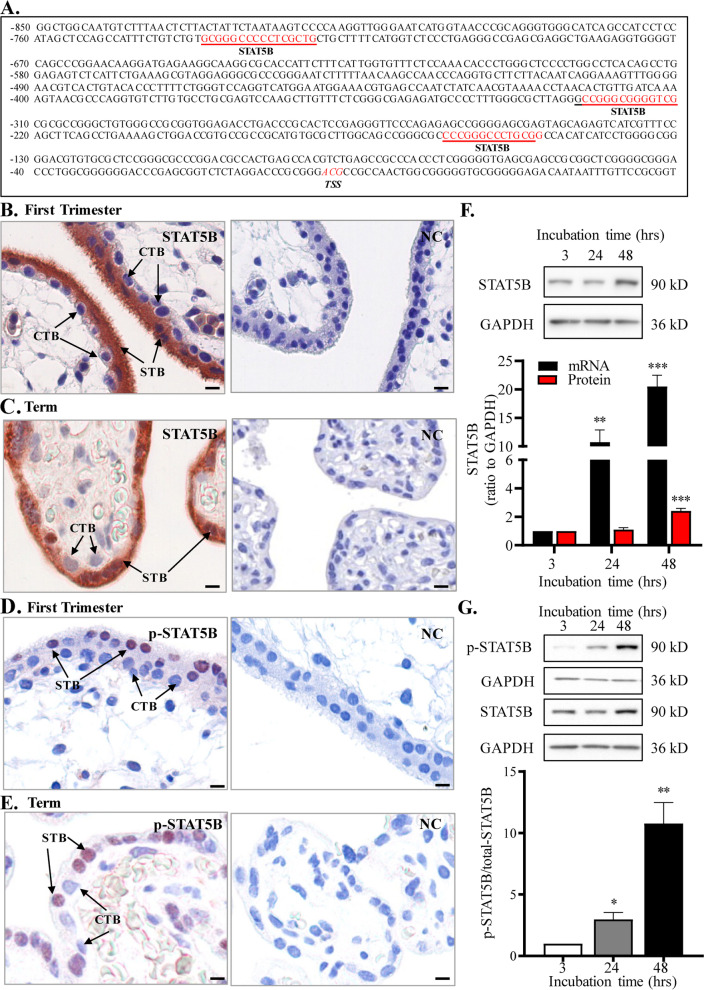
Increased STAT5B expression and phosphorylation during syncytialization of human trophoblasts. **A** The putative STAT5B binding sites at the *ADAM12* promoter revealed by in silico analysis. TSS, transcription start site. **B**–**E** Immunohistochemical staining showed that total STAT5B and phosphorylated STAT5B were mainly localized in the syncytiotrophoblast (STB) but not the cytotrophoblast (CTB) of human placental villi at both early and term pregnancies. Total STAT5B was localized in both cytoplasm and nucleus, while phosphorylated STAT5B was localized mainly in the nucleus of syncytiotrophoblast. Non-immune serum served as negative control (NC). Scale bars, 20 μm. **F** Increased *STAT5B* mRNA (*n* = 6) and protein (*n* = 7) abundance during syncytialization of cultured human trophoblasts. (G) Increased STAT5B phosphorylation during syncytialization of cultured human trophoblasts (*n* = 4). Top panels of F and G are representative immunoblots. Data are means ± SEM. *P < 0.05, **P < 0.01, ***P < 0.001 vs 3 hrs

**Fig. 4:**
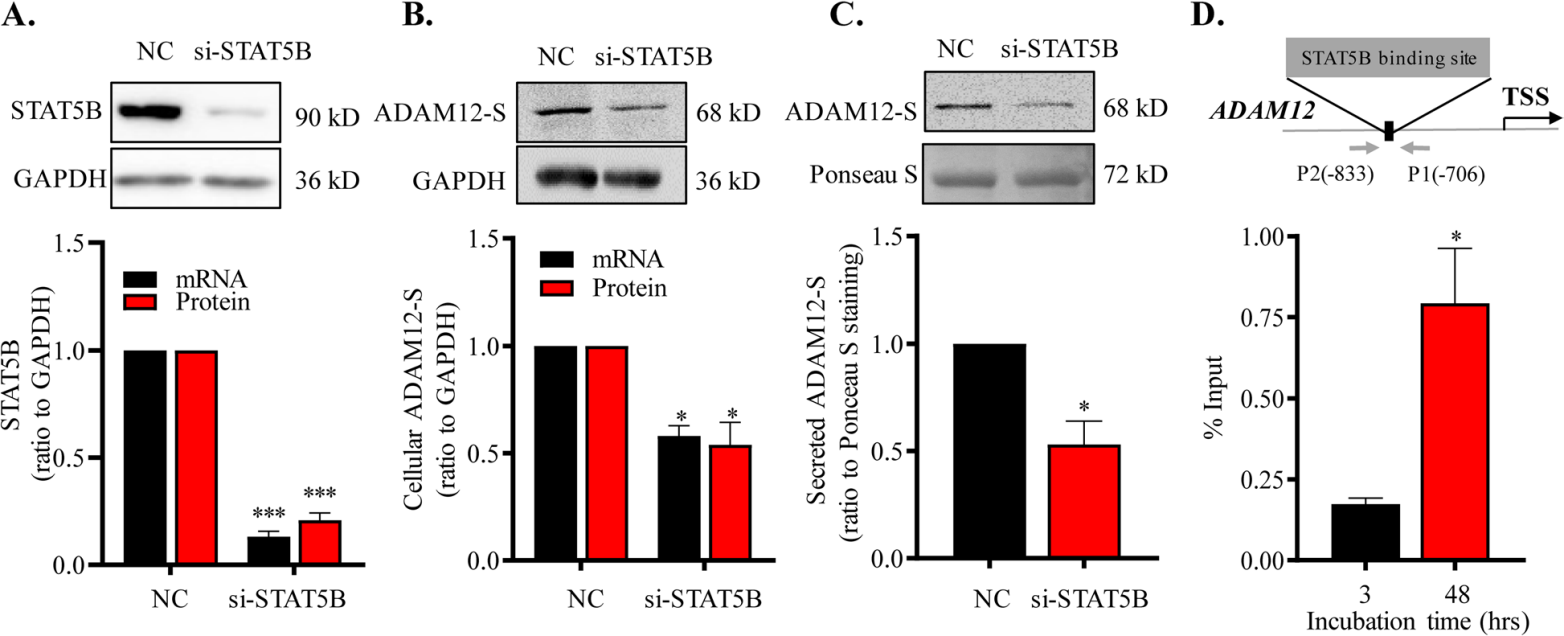
Role of STAT5B in the regulation of ADAM12-S expression during syncytialization of human trophoblasts. **A**–**C** siRNA-mediated knockdown of STAT5B expression increased the abundance of *ADAM12S* mRNA (*n* = 4) and protein in cultured trophoblasts (*n* = 6) as well as secreted ADAM12-S in trophoblast culture medium (*n* = 3). Randomly scrambled siRNA served as negative control (NC). Top panels of **A**–**C** are representative immunoblots. **D** Increased enrichment of STAT5B at *ADAM12* promoter upon syncytialization of cultured trophoblast (*n* = 4). Top panel of **D** illustrates aligning positions of primers used in ChIP assay. TSS, transcription start site. Data are means ± SEM. *P < 0.05, ***P < 0.001 vs NC or 3 hrs

**Fig. 5:**
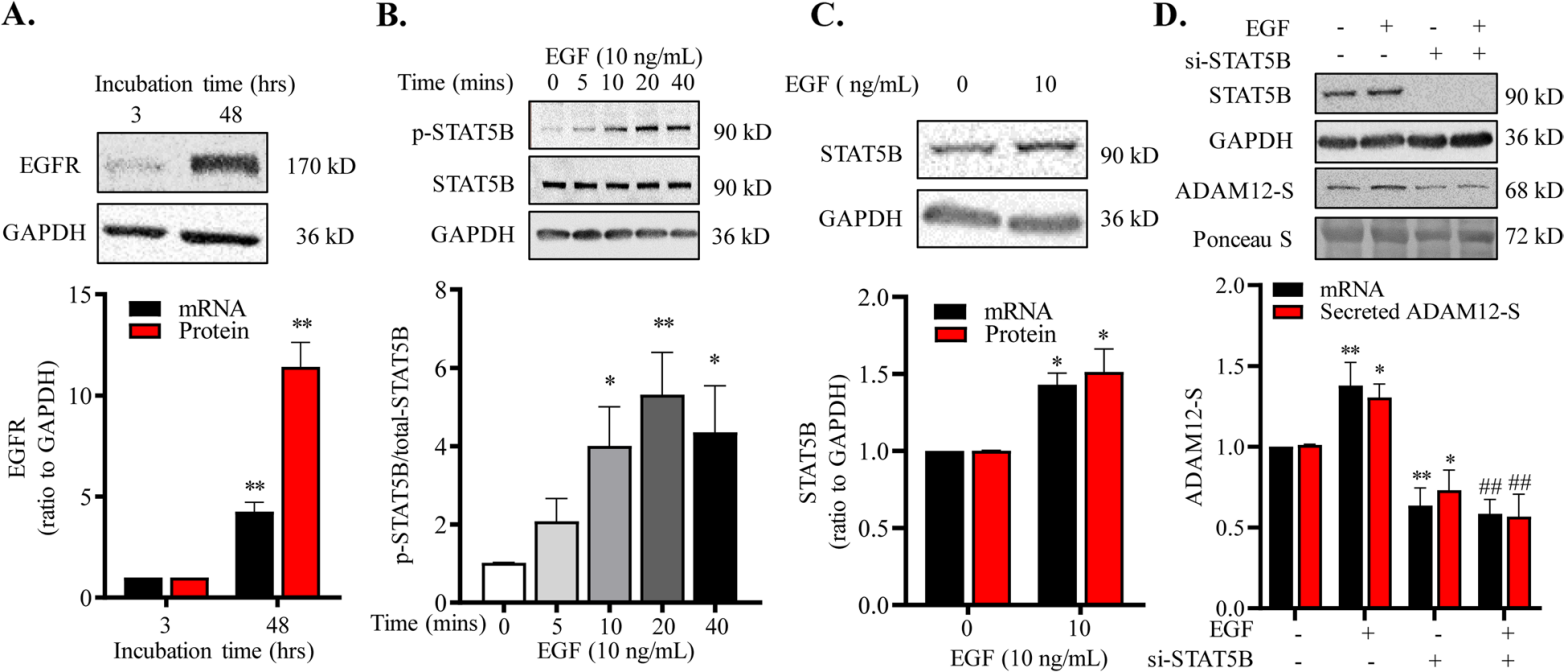
Role of EGF/STAT5B pathway in the induction of ADAM12-S expression during syncytialization of human trophoblast. **A** Increased *EGFR* mRNA and protein abundance during syncytialization (*n* = 4). **B** Time course of EGF (10 ng/mL) on STAT5B phosphorylation in syncytiotrophoblasts (*n* = 4). **C** Effects of EGF (10 ng/mL, 24 hrs) on *STAT5B* mRNA (*n* = 3) and protein abundance in syncytiotrophoblasts (*n* = 4). **D** siRNA-mediated knockdown of STAT5B attenuated basal and EGF (10 ng/mL)-induced *ADAM12S* mRNA expression (*n* = 5) and ADAM12-S secretion (*n* = 5) in cultured trophoblasts. Randomly scrambled siRNA served as negative control (NC). Top panel of each figure is the representative immunoblot. Data are means ± SEM. *P < 0.05, **P < 0.01 vs corresponding control; ^##^*P* < 0.01 vs EGF-treated group

In silico analysis of the promoter of *ADAM12* gene revealed multiple putative STAT5B binding sites (Fig. 3A). Immunohistochemical staining showed that both STAT5B and phosphorylated STAT5B were found mainly in the syncytial layer of human placental villi at both early and term gestation (Fig. 3B–E). Unlike the distribution of STAT5B in both cytoplasm and nucleus, phosphorylated STAT5B was found mainly in the nuclei of the syncytial layer (Fig. 3B–E). Consistently, syncytialization of cultured human placental cytotrophoblasts was accompanied by significant increases in *STAT5B* mRNA and protein as well as phosphorylated STAT5B abundance (Fig. 3F, G). Moreover, siRNA-mediated knock-down of STAT5B significantly decreased *ADAM12S* mRNA and protein in trophoblasts and secreted ADAM12-S in the culture medium of trophoblasts (Fig. 4A–C). ChIP assay revealed that the enrichment of STAT5B was significantly increased at *ADAM12* promoter upon syncytialization (Fig. 4D). Further investigation of the upstream signal for STAT5B activation revealed that EGF played an important role. Syncytialization was accompanied by increased *EGFR* mRNA and protein abundance (Fig. 5A). EGF treatment (10 ng/mL) of syncytiotrophoblasts for short time (less than 40 mins) increased only STAT5B phosphorylation but not its expression (Fig. 5B). However, longer time treatment with EGF (10 ng/mL, 24 hrs) significantly increased STAT5B expression at both mRNA and protein levels (Fig. 5C). Knock-down of STAT5B with siRNA transfection significantly attenuated not only basal but also EGF (10 ng/mL, 24 hrs)-induced ADAM12-S expression and secretion by syncytiotrophoblasts (Fig. 5D).

### Effects of intraperitoneal administration of EZH2 inhibitor on fetoplacental weights and H3K27me3, ADAM12S, Hsd11b2 abundance in the placenta, and ADAM12-S in maternal blood of the mouse

**Fig. 6:**
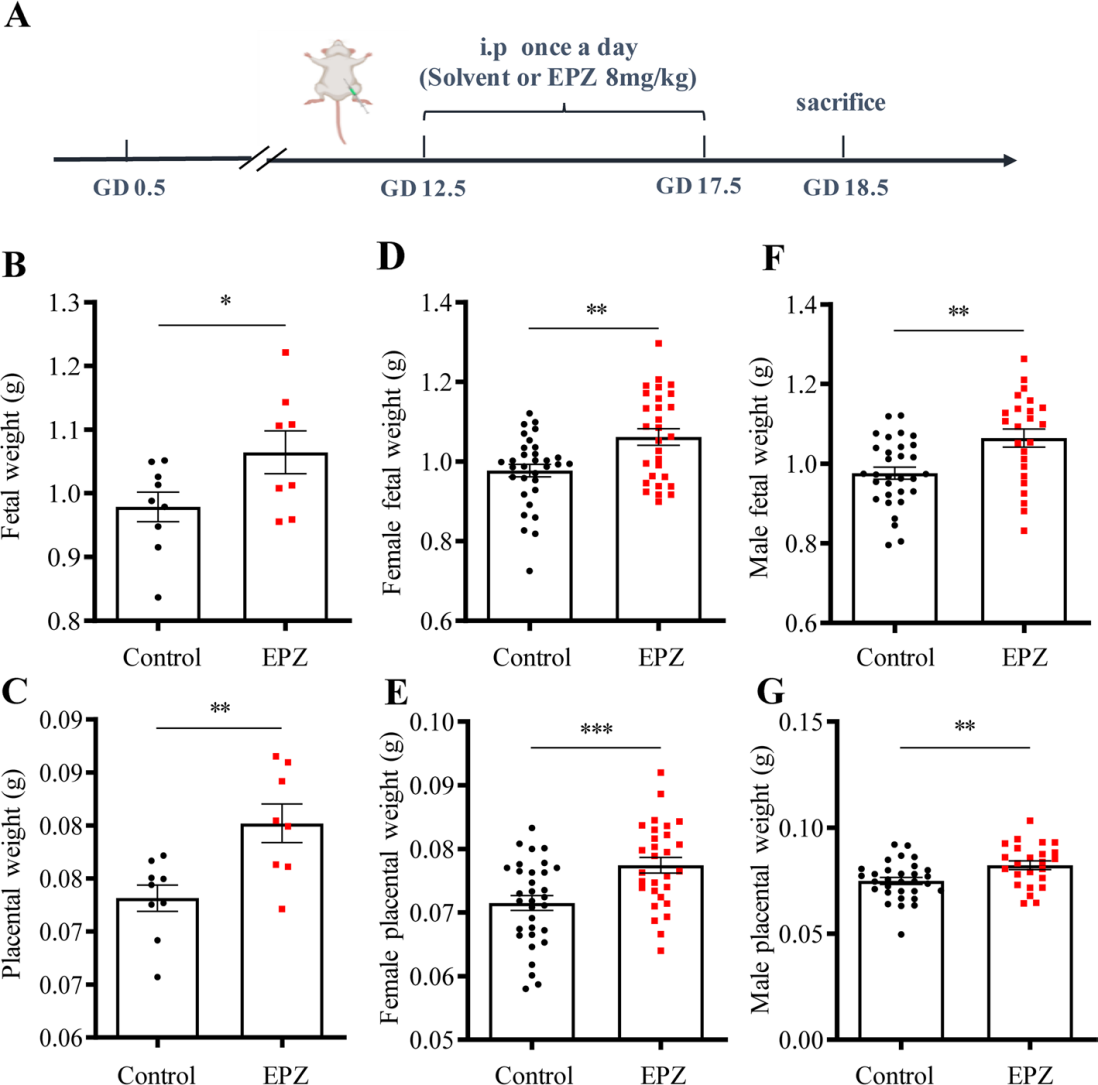
Effect of intraperitoneal administration of EZH2 inhibitor on fetoplacental weights in the mouse. **A** Time-line illustrating experimental procedures in pregnant mice. i.p., intraperitoneal injection. **B**–**G** Administration of EZH2 inhibitor EPZ005687 (EPZ) (8 mg/kg BW) increased the average fetal (**B**) and placental (**C**) weights per litter (control, *n* = 9 dams; EPZ, n = 8 dams) as well as the fetal (**D** and **F**) and placental (**E** and **G**) weights of each sex. Female fetuses: control group, *n* = 32; EPZ group, *n* = 29; male fetuses: control group, *n* = 31; EPZ group, *n* = 24. Data are means ± SEM. *P < 0.05, **P < 0.01, ***P < 0.001 vs control group

**Fig. 7:**
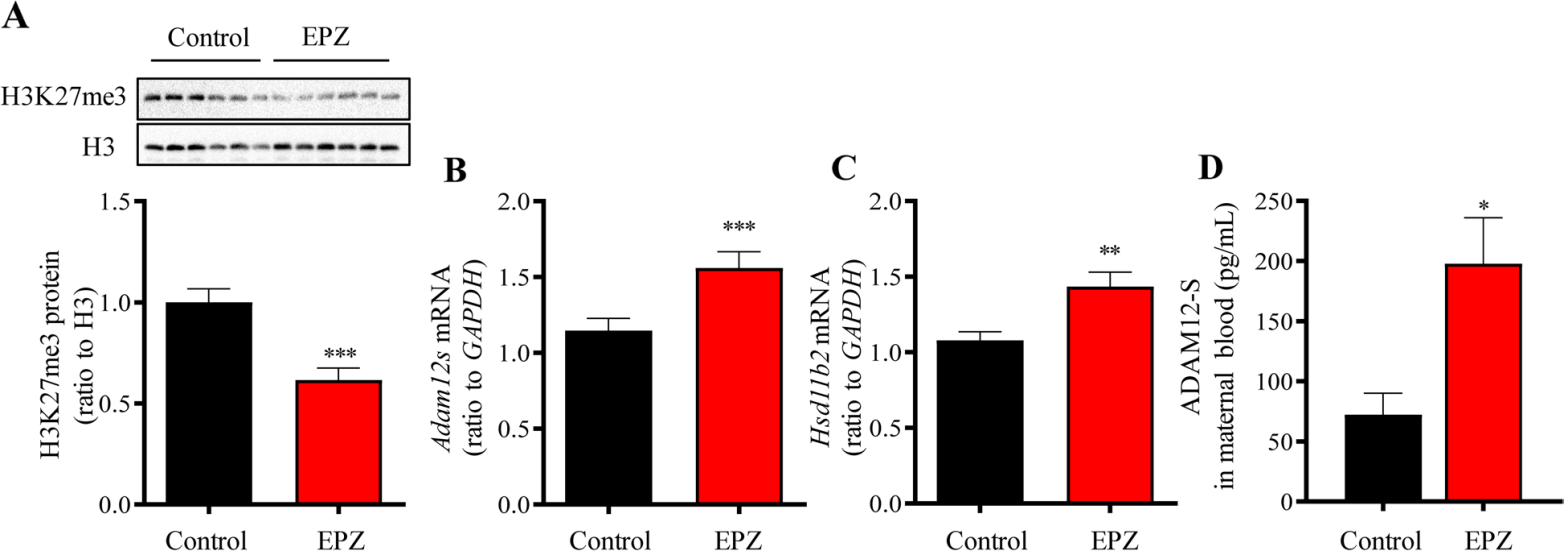
Effect of intraperitoneal administration of EZH2 inhibitor on placental H3K27me3, *ADAM12*, *Hsd11b2* abundance, and maternal blood ADAM12-S level in the mouse. **A** EPZ administration (8 mg/kg BW) decreased the abundance of placental H3K27me3 (control group, *n* = 24; EPZ group, *n* = 24). Top panel is the representative immunoblot. **B**, **C** EPZ administration (8 mg/kg BW) increased the abundance of placental *Adam12s* and *Hsd11b2* mRNA (control group, *n* = 63; EPZ group, *n* = 53). **D** EPZ administration (8 mg/kg BW) increased ADAM12-S level in maternal blood (control group, *n* = 7; EPZ group, *n* = 6). Data are means ± SEM. *P < 0.05, **P < 0.01, ***P < 0.001 vs control group

Intraperitoneal injection of EPZ005687 (EPZ) (8 mg/kg BW), a specific EZH2 inhibitor, during GD12.5–17.5 significantly increased the average placental and fetal weights per litter irrespective of sex on GD18.5 (Fig. 6A–C). Further analysis of potential sex effects on fetoplacental weights showed that EPZ administration significantly increased fetal and placental weights in both sexes (Fig. 6D–G). Western blotting analysis showed that H3K27me3 abundance was decreased significantly in the placenta by EPZ administration (Fig. 7A). Analysis with qRT-PCR showed that the abundance of *Adam12s* and *Hsd11b2* mRNA was significantly increased in the placenta by EPZ administration (Fig. 7B, C). Analysis with ELISA revealed that EPZ administration significantly increased ADAM12-S levels in maternal blood from 72.11 ± 17.96 pg/mL of the vehicle control group to 197.79 ± 3 8.31 pg/mL of the EPZ group (Fig. 7D).

## Discussion

EZH2 is one of the core components of polycomb repressive complex 2 (PRC2) and is responsible for the methyltransferase activity of PRC2. EZH2 generates di/trimethylated lysine 27 in histone 3 thus condensing chromatin structure to silence gene transcription [1]. It has been demonstrated that EZH2 is required for stable repression of homeotic selector genes and for the establishment of embryonic stem cells during early embryo development [2, 3]. Blastocysts deficient in *Ezh2* fail to gastrulate and outgrow after implantation, thus *Ezh2*-deficiency is lethal at the early stage of mouse development [2, 3]. Naturally, EZH2 in blastocysts is up-regulated upon fertilization as well as at the peri-implantation stage [2]. The outer trophectoderm layer of blastocyst gives rise to extra-embryonic trophoblast cells including villous cytotrophoblasts, the progenitor of syncytiotrophoblast. Interestingly, as shown in the present study as well as in the previous study [4], the expression of EZH2 remains high in cytotrophoblasts at both early and term gestation, but declines dramatically when cytotrophoblasts fuse to form syncytiotrophoblasts [4]. Our previous work has demonstrated that the decline in EZH2-mediated H3K27me3 upregulates 11β-HSD2 expression during syncytialization of cultured human cytotrophoblasts so that expression of the enzyme maintaining the placental glucocorticoid barrier is enhanced for the protection of normal fetal growth [4, 5]. The present study also showed that 11β-HSD2 was localized mainly in the syncytial layer of the placental villi, and indeed, our in vivo study in the mouse showed that inhibition of EZH2 significantly increased 11β-HSD2 expression in the placenta. In addition to 11β-HSD2, we provided evidence that removal of the repression by EZH2-mediated H3K27me3 upregulated ADAM12-S, another enzyme pertinent to fetoplacental growth, during syncytialization. More importantly, inhibition of EZH2 increased fetoplacental weights. These findings suggest that the role of EZH2-mediated H3K27me3 may switch from regulation of blastocyst differentiation at the peri-implantation stage to regulation of fetoplacental growth in later gestation through upregulation of at least *ADAM12* and *HSD11B2* expression in the placenta.

Although other proteases such as pregnancy-associated plasma protein A2 (PAPPA2) may also be involved in IGFBP3 cleavage [31], it appears that ADAM12-S accounts for the majority IGFBP3 proteolytic activity in maternal blood in pregnancy [17–21]. Given that IGFBP3 binds over 95% IGFs in the circulation [10, 13, 14] and the placenta is a major source of ADAM12-S [17], it is logical to assume that the upregulation of ADAM12-S in the placenta upon removal of EZH2-mediated H3K27me3 is essential for fetoplacental growth. However, it should also be acknowledged that there are other IGFBPs which regulate IGF bioavailability as well, particularly those synthesized locally in gestational tissues [32–35]. At the present stage, we are unclear whether EZH2-mediated H3K27me3 is also involved in the regulation of the expression of those IGFBPs and their related proteases. Given the broad range of EZH2-mediated epigenetic modulation of gene expression, it is possible that *ADAM12* and *HSD11B2* may not be the sole genes upregulated by inhibition of EZH2-mediated H3K27me3 in terms of fetoplacental growth regulation.

Of interest, it has been reported that the female placenta presents higher EZH2 activity and H3K27m3 levels than the male placenta because of structural stabilization of EZH2 by the highly expressed O-linked N-acetylglucosamine transferase in the female placenta [36]. However, we failed to observe any sexually dimorphic effects of EZH2 inhibition on fetoplacental weights. Nevertheless, growth-regulating factors derived from individual placenta might enter maternal circulation, which would, in turn, affect other fetuses of the same litter irrespective of sex.

Lifting EZH2-mediated H3K27me3 is known to loosen chromatin structure so that the transcription factor can access to the corresponding gene promoter to alter its transcription. However, very little is known about the transcription factor driving *ADAM12* expression. It was reported that Z-DNA silencer could inhibit *ADAM12* expression by binding to the 5′-UTR of *ADAM12* in cells with low *ADAM12* expression but not in cells with high *ADAM12* expression such as JEG-3 cells, a human choriocarcinoma trophoblast cell line [37], indicating that this mechanism is unlikely to operate during syncytialization of trophoblasts. It was also shown that E2F transcription factor 1 (E2F1) drives *ADAM12* expression in small cell lung cancer cells [38]. However, our previous study showed that the transcriptional activity of E2F1 is inactivated by the retinoblastoma protein (pRB) upon syncytialization of cytotrophoblasts [4], suggesting that it is unlikely that E2F1 is a transcription factor driving *ADAM12* expression during syncytialization. Here, we demonstrated for the first time that STAT5B is a transcription factor driving *ADAM12* expression in human placental trophoblasts. In addition, we found that EGF was at least one of the important upstream signals that stimulate *ADAM12* expression via activation of STAT5B in placental trophoblasts. Our findings are in line with the previous reports showing that multiple growth factors, including EGF, utilize STAT5B as a transcription factor [25–27]. Notably, EGF in maternal blood and EGFR in the placenta are increased in pregnancy [39–41]. The present study also found that the expression of EGFR increased during syncytialization. As one of the most highly expressed growth factor receptors in the placenta [42, 43], EGFR distributes mainly on the microvillous membrane rather than the basolateral membrane of the syncytial layer [44], indicating that EGF of the maternal circulation may play an important role in the regulation of fetoplacental growth [45]. EGF has been reported to promote fetoplacental growth through multiple pathways [46–48]. Here, we demonstrated that EGF may also regulate fetoplacental growth by increasing ADAM12-S expression via activation of STAT5B in the placenta.

## Conclusions

We have demonstrated in this study that EZH2-mediated H3K27me3 represses ADAM12-S expression in trophoblasts before syncytialization, and this repression is lifted upon syncytialization. Removal of EZH2-mediated H3K27me3 may allow access of STAT5B to the gene encoding ADAM12-S following activation by growth factors such as EGF, which results in increased ADAM12-S expression in the placenta. Given the well-recognized role of ADAM12-S in IGFBP3 cleavage, IGF-1/2 bioavailability may thus be increased to stimulate fetoplacental growth. Our studies suggest that the role of EZH2-mediated H3K27me3 may switch from cell lineage specification at the early blastocyst stage to regulation of fetoplacental growth in later gestation through regulation of at least *ADAM12* and *HSD11B2* expression in the placenta. However, it should also be borne in mind that there may be additional EZH2-regulated genes involved in the regulation of fetoplacental growth, given the broad range of genes regulated by EZH2-mediated H3K27me3.

## Data Availability

The original data and materials presented in the study are available from the corresponding authors upon reasonable request.

## Abbreviations

EZH2: Enhancer of zeste homolog 2
H3K27me3: Trimethylation of histone 3 at lysine27
11β-HSD2: 11β-hydroxysteroid dehydrogenase 2
IGFs: Insulin-like growth factors
IGF-1R: IGF receptor type 1
IGFBPs: Insulin-like growth factor binding proteins
ADAM12-S: A short secretory isoform of a disintegrin and metalloprotease 12
STAT5B: Signal transducer and activator of transcription 5B
EGF: Epidermal growth factor
EGFR: EGF receptor
hCG: Human chorionic gonadotropin
PRC2: Polycomb repressive complex 2
PAPPA2: Pregnancy-associated plasma protein A2
E2F1: E2F transcription factor 1
pRB: Retinoblastoma protein
PBS: Phosphate buffer solution
DMEM: Dulbecco’s modified Eagle’s medium
FBS: Fetal bovine serum
HE: Hematoxylin–eosin
qRT-PCR: Quantitative real-time polymerase chain reaction
siRNA: Small interfering RNA
cDNA: Complementary DNA
GAPDH: Glyceraldehyde 3-phosphate dehydrogenase
RIPA: Radioimmunoprecipitation assay
ChIP: Chromatin immunoprecipitation
GD: Gestational day
SEM: Standard error of mean
SDS: Sodium dodecyl-sulfate
